# The long noncoding RNA H19 promotes tamoxifen resistance in breast cancer *via* autophagy

**DOI:** 10.1186/s13045-019-0747-0

**Published:** 2019-07-24

**Authors:** Ji Wang, Shuduo Xie, Jingjing Yang, Hanchu Xiong, Yunlu Jia, Yulu Zhou, Yongxia Chen, Xiaogang Ying, Cong Chen, Chenyang Ye, Linbo Wang, Jichun Zhou

**Affiliations:** 1 Department of Surgical Oncology, Sir Run Run Shaw Hospital, Zhejiang University School of Medicine, No.3 Eastern Qingchun Road, Hangzhou, 310016 Zhejiang China; 2 Biomedical Research Center and Key Laboratory of Biotherapy of Zhejiang Province, Hangzhou, 310016 Zhejiang China; 3 Cancer Institute (Key Laboratory of Cancer Prevention & Intervention, National Ministry of Education), Second Affiliated Hospital, Zhejiang University School of Medicine, Hangzhou, 310009 China

**Keywords:** LncRNA H19, Autophagy, Methylation, Beclin1, Breast cancer, Tamoxifen resistance

## Abstract

**Background:**

Tamoxifen resistance remains a clinical challenge for hormone receptor-positive breast cancer. Recently, dysregulations in autophagy have been suggested as a potential mechanism for tamoxifen resistance. Although the long noncoding RNA H19 is involved in various stages of tumorigenesis, its role in tamoxifen resistance remains unknown. Here, we assessed the role of H19 in the development of tamoxifen-resistant breast cancer.

**Methods:**

Quantitative real-time PCR analyzed expression of H19 in tamoxifen-resistant breast cancer tissues. Knockdown of H19 was used to assess the sensitivity to tamoxifen in vitro and in vivo. Both knockdown and overexpression of H19 were used to analyze the status of autophagy. Real-time quantitative methylation-specific polymerase chain reaction, chromatin immunoprecipitation, immunofluorescence, and Western blot were used to explore the tamoxifen resistance mechanism of H19.

**Results:**

In this study, we observed that the expression of H19 was substantially upregulated in tamoxifen-resistant breast cancer cell line and tumor tissues, and knockdown of H19 enhanced the sensitivity to tamoxifen both in vitro and in vivo. Furthermore, knockdown of H19 significantly inhibited autophagy in MCF7 tamoxifen-resistant (MCF7/TAMR) cells. Conversely, overexpression of H19 promoted autophagy. Interestingly, overexpression of H19 in MCF7 tamoxifen-sensitive cells could recapitulate tamoxifen resistance. Moreover, an increase in methylation in the promoter region of Beclin1 was observed in MCF7/TAMR-shH19 cells. In the double knockdown groups, both shH19+shSAHH and shH19+shDNMT3B rescued the Beclin1 promoter region methylation levels and reactivated autophagy functions. A chromatin immunoprecipitation assay further validated that DNMT3B binds to the Beclin1 promoter region and the knockdown of H19 increases this binding.

**Conclusions:**

Our findings demonstrate that H19 induces autophagy activation *via* the H19/SAHH/DNMT3B axis, which could contribute to tamoxifen resistance in breast cancer.

**Electronic supplementary material:**

The online version of this article (10.1186/s13045-019-0747-0) contains supplementary material, which is available to authorized users.

## Background

Breast cancer is the most frequently diagnosed malignancy and the second leading cause of cancer mortality in females worldwide [1]. Approximately 70% of breast cancer patients are estrogen receptor (ER)-positive. Tamoxifen, an antiestrogen, competitively inhibits the binding of estrogen to the ER and blocks the ER-mediated stimulation signal [2]. Five years of tamoxifen adjuvant therapy has been shown to safely reduce 15-year risks of breast cancer recurrence and death [3]; however, a substantial group of patients was shown to eventually develop resistance (de novo or acquired) to tamoxifen [3, 4]. Although many molecular mechanisms of tamoxifen resistance have been revealed, including mutations in the *ESR1* gene and the activation of alternative growth pathways, such as ERBB2/HER2, EGFR, IGF1R, and cyclin D1/CDK4/6 pathways [5–7], it remains necessary to gain an improved understanding of the potential mechanisms of tamoxifen resistance.

Autophagy is a cellular process through which intracellular misfolded proteins and malfunctioning organelles are targeted to lysosomes or vacuoles for degradation [8, 9]. Recent studies have shown that autophagy is also a potential mechanism for tamoxifen resistance. For instance, overexpression of Beclin1, the key mediator of autophagy, desensitizes estrogen-induced signaling, contributing to the development of tamoxifen resistance in ER-positive breast cancers [10]. Inhibition of autophagy genes, such as atg5, atg7, and Beclin1, results in resensitization of tamoxifen-resistant breast cancer cells [11, 12]. The autophagy inhibitors 3-methyladenine (3-MA) and chloroquine (CQ) have been used to restore tamoxifen sensitivity in tamoxifen-resistant cancer [13, 14]. However, the underlying mechanism by which autophagy mediates tamoxifen resistance in breast cancer remains to be elucidated.

Long noncoding RNAs (lncRNAs) (> 200 bp) have been shown to participate in a variety of biological processes, including tamoxifen resistance [15–19]. For example, the lncRNA *HOTAIR* enhances ligand-independent ER function and contributes to tamoxifen resistance [20]. The lncRNA DSCAM-AS1 facilitates estrogen-independent oncogenicity, which could also potentially promote tamoxifen resistance [21]. H19 lncRNA (H19) is an imprinting lncRNA that is exclusively transcribed from the maternally inherited allele [22, 23]. H19 plays important roles in proliferation, metastasis, chemoresistance, and stem cell maintenance of breast cancer cells [24–27]. Recent research has shown that H19 is more abundant in ER-positive breast cancer than in ER-negative breast tumor tissues [28]. Moreover, some evidence has indicated that blocking ERs in luminal progenitor cells results in downregulated H19 expression and smaller colony formation, similar to the H19-knockdown phenotype [29]. However, the role of H19 in the development of tamoxifen resistance remains vague.

In this study, we showed that H19 promoted tamoxifen resistance in ER-positive breast cancer cells and autophagy, which occurs *via* downregulation of methylation in the promoter of Beclin1 by the H19/SAHH/DNMT3B axis. This novel molecular mechanism for tamoxifen resistance may serve as a promising biomarker for overcoming tamoxifen resistance.

## Methods

### Cell culture

The tamoxifen-sensitive human breast cancer cell line MCF7 was obtained from the American Type Culture Collection (ATCC, HTB-22). Tamoxifen-resistant cells (MCF7/TAM) were established by culturing MCF7 cells in medium with 1 μM tamoxifen citrate salt (Sigma-Aldrich, T9262-1G) over 6 months, as previously described [30, 31]. All of the cells were cultured in RPMI 1640 medium with 10% fetal bovine serum (FBS) at 37 °C in the presence of 5% CO_2_.

### Plasmid, siRNA, and transfection

To generate H19-knockdown MCF7/TAM cells, we purchased synthesized target sequences for scrambled siRNA and H19 siRNA from Thermo Fisher (4390771). MCF7/TAM cells were transfected with 1 μg of LC3-EGFP-mCherry plasmid using Lipofectamine® 3000 transfection reagent (Invitrogen, L3000001). The stable cell line was selected using G418 sulfate antibiotic (Calbiochem, 509290).

### Lentivirus-mediated transduction

According to previous studies and results from NCBI BLAST, we used the following target sequences: H19 (5-′CAGCCCAACATCAAAGACA-3′), SAHH (5′-ACAACCTCTACAAGATGAT-3′), DNMT3B (5′-AGATGACGGATGCCTAGAG-3′), and scrambled sequence (5′-TTCTCCGAACGTGTCACGT-3′) [32, 33]. These sequences were cloned into GV248/GV307 vectors (GeneChem, Shanghai, China). The plasmid H19 was purchased from GeneChem. All plasmids were transfected into 293T cells, together with the Lentivector Expression System (GeneChem, Shanghai, China), to produce lentiviruses. These specific shRNAs were packaged into lentiviruses by Genechem Inc. For the infection, target cells were cultured at a density of 100,000 cells per well in 6-well plates, cocultured with 2.5E+6 Tu virus in the presence of 5 mg/ml polybrene and standard medium for 13 h, and then the medium was changed to fresh medium. After 72 h of transfection, the cells were selected *via* incubation with 10 μg/ml puromycin for 1 week. We used qRT-PCR and/or Western blotting to confirm the expression of the target genes.

### Colony formation

Cells were seeded at a density of 300–1000 cells per well in 6-well plates in standard medium. After 24 h, the medium was changed to medium with tamoxifen, and the medium was regularly replaced every 2 days. After 2 weeks, live cells were stained using crystal violet.

### Cell viability

Cells were seeded at a density of 3000 cells per well in 96-well plates (in triplicate). Tamoxifen was added (day 0) 24 h after cell seeding and redosed along with the medium changes every 48 h after the first dose. A cell counting kit-8 (CCK-8, Dojindo, Japan, CK04) was used to analyze the number of viable cells from day 0 to day 5. Following the manufacturer’s instructions, 10 μl of CCK-8 reagent was mixed with 100 μl of normal medium in each well. After incubating for 4 h at 37 °C, the absorbance was recorded at a wavelength of 450 nm.

### Analysis of autophagy by flow cytometry

Cells were seeded in 6-well plates for 24 h, then changed into fresh media containing 10 μM tamoxifen and incubated for 24 h. The cells were washed, and the autophagic vacuoles were quantified using a cyto-ID autophagy detection kit (Enzo, ENZ-51031) according to the manufacturer’s instructions. The signals of labeled autophagic vacuoles were analyzed using a flow cytometer with an FL1 (488 nm excitation, green) channel.

### Fluorescent confocal microscopy

MCF7/TAMR cells stably expressing tandem mCherry-EGFP-LC3 (described previously) were further infected with shCtrl, shH19, shH19+shSAHH, or shH19+shDNMT3B and selected using puromycin for 1 week. Then, the cells were seeded on a coverglass for growth and cultured in medium with 10 μM tamoxifen. After 24 h, the cells were fixed, stained with DAPI (300 nM), and then examined using a confocal microscope (Nikon A1 Ti).

### Apoptosis analysis

Floating and attached cells were collected, and apoptosis was measured using a FITC Annexin V Apoptosis Detection Kit I (BD Biosciences, 556547) according to the manufacturer’s protocol. Briefly, a total of 50,000 cells per replicate (three independent experiments) were washed and incubated with FITC Annexin V and PI, and the green fluorescence of annexin V and red fluorescence of PI were analyzed by flow cytometry using FL1 (488 nm excitation, green) and FL3 (585 nm excitation, red) channels. A minimum of 10,000 events was collected for each sample.

#### Cell cycle analysis

Cells were collected at 48 h after transfection with an siRNA, and then washed twice with PBS, and the Cell Cycle Staining Kit (MULTISCIENCES, CCS012) was used following the manufacturer’s instructions. Cell cycle analyses were performed by flow cytometry (Accuri model C6).

### RNA extraction and quantitative real-time PCR

Total RNA was extracted using an E.Z.N.A.® Total RNA Kit I (OMEGA, Norcross, USA). The RNA concentration was measured using a Nanodrop 2000c (Thermo Scientific, USA). One microgram of RNA was reverse transcribed using a HiFiScript cDNA Synthesis Kit (CWBIO, Beijing, China) with random primers. The expression of H19, SAHH, DNMT3B, Beclin1, and tubulin genes was determined by real-time PCR using SYBR Green Master Mix (CWBIO, Beijing, China) with the primers (Sangon Biotech, Shanghai, China) listed in Additional file 2: Table S2. A final volume of 25 μl was used for qPCR in an ABI 7500 Real-Time PCR System (Applied Biosystems, USA). The amplification conditions were 95 °C for 5 min, followed by 40 cycles at 95 °C for 15 s, 60 °C for 30 s, and 72 °C for 30 s. Tubulin was used to correct the difference in template input. The relative RNA expression was calculated using the 2^−ΔCT^ method.

### Real-time quantitative methylation-specific polymerase chain reaction analysis

Genomic DNA was extracted using a Quick-gDNA MicroPrep Kit (Zymo, D3021) and eluted in 20 μl of DNase-free water. Then, 500 ng of DNA was bisulfited using a EZ DNA Methylation-Gold Kit (Zymo, D5006). RT-qPCR was performed using ChamQ SYBR (Vazyme) in the ABI 7500 Real-Time PCR System (Applied Biosystems, USA). The PCR primers for methylated DNA are listed in Additional file 2: Table S1. PCR was performed by initial denaturation at 95 °C for 30 s, followed by 40 cycles at 95 °C for 10 s and 60 °C for 30 s. Specificity was verified by melting curve analysis. The Ct values of each sample were used for subsequent data analysis. Albumin DNA was used as a loading control for all quantitative methylation-specific polymerase chain reaction (QMSP) data normalization.

### Chromatin immunoprecipitation assay

A SimpleChIP® Enzymatic Chromatin IP Kit (Magnetic Beads) (Cell Signaling Technology, #9003) was used following the manufacturer’s instructions. Briefly, crosslinking was performed by fixing 4 × 10^6^ cells with 37% formaldehyde for 10 min at room temperature, and the crosslinking reaction was quenched by glycine. Sonication and enzymatic digestion were used to digest chromatin from the lysed cells. Chromatin was then immunoprecipitated using anti-DNMT3B (Cell Signaling Technology, D7070) and standard rabbit-IgG antibodies. Next, chromatin immunoprecipitation (ChIP)-enriched DNA was amplified using PCR, and the primer sets were designed as follows: Beclin1: 5′-GGTCAGCGAGACCCTTGGAA-3′ (sense) and 5′AGAATTATATCACCAAAGCTGCCC-3′ (anti-sense). The PCR products were loaded onto 2% agarose gels and observed using ultraviolet light.

### Western blot analysis

A total of 1 × 10^6^ cells were plated in each 60-mm dish and allowed to attach for 24 h before the treatments. After the treatments, the cells were lysed using RIPA buffer (Pierce, Rockford, USA) mixed with a protease inhibitor cocktail. The concentrations of proteins were determined using a Bio-Rad protein assay kit II (Bio-Rad Laboratories, 500-0002EDU). A total of 15 μg of protein from each sample was mixed with 5× Lane Marker Reducing Sample Buffer (Pierce, Rockford, USA), separated on a 12% SDS-polyacrylamide gel, then transferred to PVDF membranes (Merck Millipore, Billerica, USA), and incubated with 1:500 to 1:1000 dilutions of primary antibodies, including GAPDH (Santa Cruz Biotechnology, sc-47724), Beclin1 (Cell Signaling Technology, 3495S), LC3 (Sigma-Aldrich, L7543), P62 (MBL International, PM045), SAHH (Santa Cruz Biotechnology, sc-271389), and DNMT3B (Cell Signaling Technology, D7070). The protein bands were stained with horseradish peroxidase-conjugated secondary antibodies (MultiSciences, GAM0072, and GAR007). The protein bands were visualized by enhanced chemiluminescence (Merck Millipore, Billerica, USA). Fold changes in the intensity of the protein signals were reported as the mean of the results from three experiments.

### Xenograft studies

Animal studies were reviewed and approved by the Ethics Committee for Animal Studies of Zhejiang University. MCF7/TAMR-Tet-shH19 xenografts were established in 5-week-old nude mice (Shanghai SLAC Laboratory Animal Corporation) by inoculating 1 × 10^7^ cells (together with 50% Matrigel, BD Biosciences) into the abdominal mammary fat pad. Tumor sizes were measured, and tumor volumes were calculated as follows: length × width^2^ × 0.5. When the tumors reached a volume of approximately 200 mm^3^ (2 to 3 weeks), mice bearing MCF7/TAMR-Tet-shH19 xenografts were randomized to −/+ doxycycline (Dox, 1 mg/mouse) and −/+ tamoxifen (50 mg/kg) treatment groups. Each group consisted of a minimum of 5 mice. The diameters of the tumors were measured every 4 days. After 22 days of treatment, all mice were euthanized, and the tumors were surgically removed. Portions of the tumors were immediately frozen in liquid nitrogen for the following extraction of RNA and protein or fixed in 10% buffered formalin for immunohistochemistry.

### Immunohistochemical analysis

All formalin-fixed and paraffin-embedded tumor sections were treated with xylene and ethanol for deparaffinization and rehydration. After blocking endogenous peroxidase with 3% H_2_O_2_ in methanol, antigen retrieval was conducted by boiling the sections in sodium citrate buffer (0.1 mM, pH 6.0) for 5 min. The sections were then blocked with goat serum (C-0005, Bioss) for 30 min and incubated overnight at 4 °C with antibodies against LC3 (1:1000, Sigma-Aldrich, L7543), P62 (1:1000; MBL International, PM045), and Beclin1 (1:50; Cell Signaling Technology, 3495S). GTvisionIII Immunohistochemical Assay Kit (HRP/DAB, rabbit/mouse-general, two-step) (GK500710, Gene Tech Shanghai) and Maye’s hematoxylin were used to detect the primary antibody and the cell nucleus. Images were acquired using a polarized light microscope (Nikon, Eclipse 80i). Two independent pathologists in our hospital analyzed the staining results.

#### Sample collection and patient characteristics

Fourteen tamoxifen-sensitive and 23 tamoxifen-resistant breast cancer tissues were surgically obtained from the Department of Surgical Oncology, Sir Run Run Shaw Hospital, Zhejiang University School of Medicine, and frozen at − 80 °C. Written informed consent was obtained from each patient. The Ethics Committee of the Sir Run Run Shaw Hospital at Zhejiang University School of Medicine approved this study.

#### Public Gene Expression Omnibus dataset analysis

Two publicly available datasets (GSE26459 and GSE28645) containing gene expression information from tamoxifen-resistant breast cancer cell lines and parental tamoxifen-sensitive cell lines were downloaded from Gene Expression Omnibus (GEO; http://www.ncbi.nlm.nih.gov/geo/). The processed data including normalization procedures were obtained from the corresponding websites, and no additional transformations were performed.

### Statistical analysis

Statistical analysis was performed by using SPSS software 22.0 version or GraphPad Prism. The significance was evaluated by the simple *t* test or two-way ANOVA for normally distributed data. Non-normally distributed data was analyzed by using a two-tailed Wilcoxon signed-rank test for matched pairs. Correlation was investigated by a two-tailed Spearman parametric correlation test. A *P* value less than 0.05 was deemed to be statistically significant.

## Results

### Autophagy facilitates tamoxifen resistance in breast cancer cell lines

**Fig. 1:**
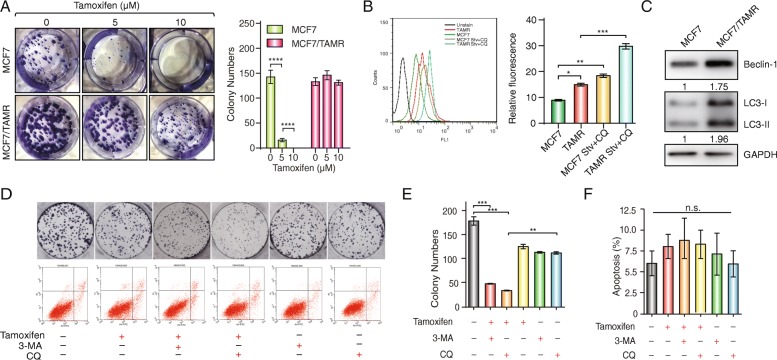
Increased autophagy promotes tamoxifen resistance. **a** Representative images of the colony formation assays using MCF7 or MCF7/TAMR cells under 0, 5, and 10 μM tamoxifen treatment for 14 days. The bar graphs show the quantification of the colony formation assay data. The data are presented as the mean ± SD of three independent experiments. Student’s *t* test was used for statistical analysis. **b** Autophagic vacuoles stimulated by 10 μM tamoxifen for 12 h in MCF7 or MCF7/TAMR cells were analyzed using a Cyto-ID autophagy detection assay. Stv: starvation. The data are presented as the mean ± SD of three independent experiments. Student’s *t* test was used for statistical analysis. **c**. Both MCF7 cells and MCF7/TAMR cells were exposed to 10 μM tamoxifen for 12 h. Western blotting was performed to examine the expression of Beclin1 and LC3. The value next to each blot is the quantification of the relative expression of the indicated band normalized to GAPDH expression. **d**. Representative images of the colony formation and cell apoptosis assays using MCF7/TAMR cells that were cultured in blank, tamoxifen, 3-MA, CQ, or tamoxifen combined with 3-MA or CQ media. **e**. Bar graphs showing the quantification of the colony formation assay data. The data are presented as the mean ± SD of three independent experiments. Student’s *t* test was used for statistical analysis. **f**. Bar graphs showing the percentage of apoptotic cells. The data are presented as the mean ± SD of three independent experiments. Student’s *t* test was used for statistical analysis. **P < 0.01, ***P < 0.001, and ****P < 0.0001 compared with the control group. n.s. indicates no significant difference

To investigate the role of autophagy in the development of tamoxifen resistance, we established a tamoxifen-resistant MCF7 cell line (MCF7/TAMR) by culturing a tamoxifen-sensitive MCF7 cell line in medium with 1 μM tamoxifen for over 6 months, as previously described [30, 31]. To confirm the establishment of the cell line, we performed a monolayer colony formation assay (Fig. 1a). After 14 days, parental MCF7 cells showed a significant decrease in survival rates with 5 or 10 μM tamoxifen treatment, whereas MCF7/TAMR cells were not affected by tamoxifen at these concentrations. Additionally, MCF7/TAMR cells had a greater IC50 value than MCF7 cells (IC50: 37.29 μM for MCF7/TAMR cells compared with 5.03 μM for MCF7 cells; *P* < 0.01) (Additional file 1: Figure S1a). To investigate whether autophagy has an impact on tamoxifen resistance, we analyzed autophagic vacuoles in both cell lines using a Cyto-ID autophagy detection assay and found that MCF7/TAMR cells showed stronger fluorescent signals, which is indicative of increased autophagy, compared with MCF7 cells (Fig. 1b). Furthermore, Western blotting indicated that MCF7/TAMR cells showed a stronger induction of autophagy than MCF7 cells based on greater expression of LC3-II (Fig. 1c). To further verify the role of autophagy in tamoxifen resistance, we used two autophagic inhibitors, 3-MA and CQ, which inhibit autophagy at upstream or downstream levels, respectively. After treating cells with 5 mM 3-MA or 5 μM CQ for 14 days (medium was changed to fresh medium every 3 days), MCF7/TAMR cells showed a decrease in resistance to 10 μM tamoxifen, indicated by a decreased number of cell colonies (Fig. 1d (upper row), e). To exclude whether the decrease in proliferation was affected by apoptosis, we compared the apoptotic levels in these groups as well. Flow cytometry results showed similar apoptotic levels among MCF7/TAMR cells cultured for 24 h in medium with blank, tamoxifen, 3-MA, CQ, or tamoxifen combined with the two autophagy inhibitors (Fig. 1d (lower row), f). These data indicated that autophagy promotes tamoxifen resistance in MCF7/TAMR cells.

### H19 is upregulated in tamoxifen-resistant breast cancer cell line and tumor tissues and promotes resistance to tamoxifen

**Fig. 2:**
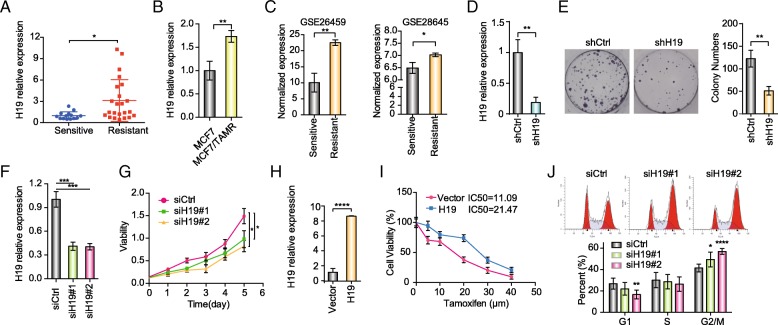
LncRNA H19 is overexpressed in tamoxifen-resistant cell line and tumor samples and facilitates tamoxifen resistance. **a** mRNA expression of H19 in 37 breast cancer tissue samples. The data are presented as the mean ± SD; *n* = 37. The Wilcoxon signed-rank test was used for statistical analysis. **b** mRNA expression of H19 in MCF7/TAMR cells and parental MCF7 cells. The data are presented as the mean ± SD of three independent experiments. Student’s *t* test was used for statistical analysis. **c** H19 expression statuses in tamoxifen-resistant and tamoxifen-sensitive MCF7 cells were obtained from GSE26459 and GSE28645. The data are presented as the mean ± SD of three independent experiments. Student’s *t* test was used for statistical analysis. **d** MCF7/TAMR cells were stably infected with the shControl (shCtrl) lentiviral vector or the shH19 lentiviral vector. The efficiency of RNA interference-mediated knockdown of target gene expression was determined by qRT-PCR analysis. The data are presented as the mean ± SD of three independent experiments. Student’s *t* test was used for statistical analysis. **e** Representative images of the colony formation assays using MCF7/TAMR cells that stably expressed shCtrl or shH19 under 10 μM tamoxifen treatment. The bar graphs show the quantification of the colony formation assay data. The data are presented as the mean ± SD of three independent experiments. Student’s *t* test was used for statistical analysis. **f** MCF7/TAMR cells were transfected with the siCtrl or the siH19. The efficiency of RNA interference-mediated knockdown of target gene expression was determined by qRT-PCR analysis. The data are presented as the mean ± SD of three independent experiments. Student’s *t* test was used for statistical analysis. **g** Under 10 μM tamoxifen treatment, the viability of MCF7/TAMR cells that transfected with siH19 or shCtrl was analyzed using a CCK-8 assay. The data are presented as the mean ± SD of three independent experiments. Student’s *t* test was used for statistical analysis. **h** MCF7 cells were stably infected with the lentiviral empty vector or the H19 overexpression lentiviral vector. The overexpression efficiency of target gene expression was determined by qRT-PCR analysis. The data are presented as the mean ± SD of three independent experiments. Student’s *t* test was used for statistical analysis. **i** H19-overexpressing MCF7 cells or parental tamoxifen-sensitive MCF7 with empty vector cultured in different concentrations of tamoxifen was analyzed using a CCK-8 assay. The data were then used to calculate the IC50 of each cell line. The data are presented as the mean ± SD of three independent experiments. Student’s *t* test was used for statistical analysis. **j** Cell cycle analysis after knockdown of H19 was conducted using flow cytometry. The data are presented as the mean ± SD of three independent experiments. Student’s *t* test was used for statistical analysis. *P < 0.05, **P < 0.01, ***P < 0.001, and ****P < 0.0001 compared with the control group

We analyzed clinical breast cancer tissues from 37 cases of breast cancer, including 14 tamoxifen-sensitive samples and 23 tamoxifen-resistant samples (cancer recurred with the adjuvant tamoxifen treatment). Overexpression of H19 was statistically more frequent in the tamoxifen-resistant group compared with the tamoxifen-sensitive group (*P* = 0.0119; Fisher’s exact test) (Fig. 2a). We further measured the level of H19 in MCF7/TAMR and parental MCF7 cell lines. The qRT-PCR results revealed that the expression level of H19 in MCF7/TAMR cells was significantly higher than parental MCF7 cells (Fig. 2b), which is consistent with previous study [34]. Similarly, GSE26459 and GSE28645 datasets from Gene Expression Omnibus (GEO) both indicated that H19 expression levels of tamoxifen-resistant cell groups were significantly upregulated compared with the tamoxifen-sensitive MCF7 cell groups (Fig. 2c) [35, 36]. To determine the role of H19 in tamoxifen resistance, we silenced H19 *via* lentivirus-mediated short hairpin RNAs (shRNAs). qRT-PCR confirmed a decrease of at least 80% in the RNA expression level of MCF7/TAMR-shH19 cells compared to that of MCF7/TAMR-control cells (Fig. 2d). Then, we evaluated the sensitivity of tamoxifen using a monolayer colony formation assay and a CCK-8 assay. The monolayer colony formation assay confirmed that MCF7/TAMR-shH19 cells formed fewer colonies than the MCF7/TAMR-control cells under the 10-μM tamoxifen treatment (Fig. 2e). Similarly, the CCK-8 assay indicated that MCF7/TAMR-shH19 cells showed a greater sensitivity than MCF7/TAMR-control cells under the 10-μM tamoxifen treatment (Additional file 1: Figure S2a). Consistently, similar cell viability results were obtained by using two independent siH19 in MCF7/TAMR cells (Fig. 2f, g). Intriguingly, we also generated stable H19-overexpressing MCF7 cells and found H19 overexpression in wild-type MCF7 can recapitulate tamoxifen resistance (Fig. 2h, i). As expected, knockdown of H19 enhanced the sensitivity of wild-type MCF7 to tamoxifen (Additional file 1: Figure S2b). To investigate the impact of H19 on cell cycle progression, we conducted cell cycle analysis using flow cytometry. Cell cycle analysis revealed that loss of H19 induced cell-cycle arrest at the G2/M phase (Fig. 2j). Collectively, we confirmed that H19 promotes tamoxifen resistance, and H19 silencing induces greater sensitivity of tamoxifen.

### H19 promotes autophagy activity in tamoxifen-resistant breast cancer

**Fig. 3:**
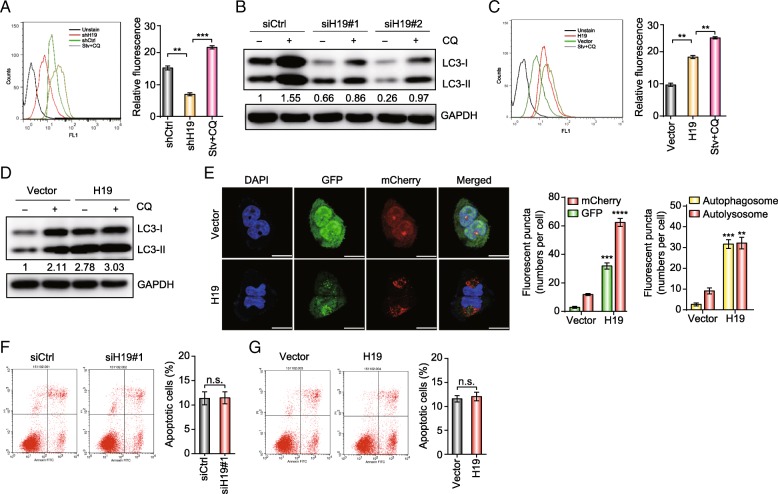
H19 lncRNA is required for the activation of autophagy. **a** Autophagic vacuoles stimulated by 10 μM tamoxifen for 12 h in MCF7/TAMR cells that stably expressed shH19 or shControl (shCtrl) were analyzed using a Cyto-ID autophagy detection assay. Stv: starvation. The bar graphs show the quantification of the relative fluorescence data. The data are presented as the mean ± SD of three independent experiments. Student’s *t* test was used for statistical analysis. **b** In MCF7/TAMR cells that stably expressed shH19 or shCtrl, LC3 expression was analyzed by Western blotting. Cells were treated without or with CQ to perform flux analysis. The value next to each blot is the quantification of the relative expression of the indicated band normalized to GAPDH expression. **c**. Autophagic vacuoles stimulated by 10 μM tamoxifen for 12 h in MCF7/TAMR cells that were stably infected with the H19 lentiviral vector or the control lentiviral vector were analyzed using a Cyto-ID autophagy detection assay. Stv: starvation. The bar graphs show the quantification of the relative fluorescence data. The data are presented as the mean ± SD of three independent experiments. Student’s *t* test was used for statistical analysis. **d** LC3 expression in MCF7/TAMR cells that were stably infected with the H19 lentiviral vector or the control lentiviral vector was analyzed by Western blotting. Cells were treated without or with CQ to perform flux analysis. The value next to each blot is the quantification of the relative expression of the indicated band normalized to GAPDH expression. **e** The distribution of autophagic vacuoles that contained mCherry-EGFP-LC3 in MCF7/TAMR cells overexpressing H19 was analyzed by confocal microscopy. The bar graphs show the quantification of the fluorescent puncta data. The data are presented as the mean ± SD of three experiments. The scale bars indicate 20 μm. Two-way ANOVA was used for statistical analysis. **f**, **g** MCF7/TAMR cells with knockdown (**f**) or overexpression (**g**) of H19 were analyzed by annexin V/PI double staining. The bar graphs show the percentage of apoptotic cells. The data are presented as the mean ± SD of three independent experiments. Student’s *t* test was used for statistical analysis. **P < 0.01, ***P < 0.001, and ****P < 0.0001 compared with the control group. n.s. indicates no significant difference

To determine the relationship between H19 and autophagy, we conducted flow cytometry and found that H19 knockdown decreased the fluorescent signal of autophagosomes compared with the control group in MCF7/TAMR cells (Fig. 3a). To determine whether inhibition of H19 influences autophagic flux in MCF7/TAMR cells, we conducted analyses using autophagy protein LC3-II and the lysosomal inhibitor CQ. As indicated by increased levels of LC3-II in the presence of CQ, decreased autophagic flux was observed in the H19 silencing groups compared with that in control group, indicating knockdown of H19 inhibited autophagic synthesis (Fig. 3b). Then, we used stable H19-overexpressing MCF7/TAMR cell line to perform flow cytometry. Ectopically, H19 expression promoted the fluorescence of autophagosomes and increased the expression of LC3-II (Fig. 3c, d). Then, we utilized the mCherry-EGFP-LC3 reporter, which can distinguish between autophagosomes and autolysosomes whose GFP signal is vulnerable to acidic conditions after autolysosome formation, whereas the mCherry signal is less affected. Therefore, the yellow puncta indicate autophagosomes, and the red puncta indicate autolysosomes in the merged figures. By overexpressing H19, the formation of both autophagosomes and autolysosomes increased (Fig. 3e), indicating increased autophagy. The decreased formations of both autophagosomes and autolysosomes were observed in H19-knockdown cells (Fig. 5a). Furthermore, we examined apoptosis by knocking down or overexpressing H19 in MCF7/TAMR cells, and the results did not show a significant difference (Fig. 3f, g).

### H19 regulates the autophagy-related gene Beclin1 *via* epigenetic regulation

**Fig. 4:**
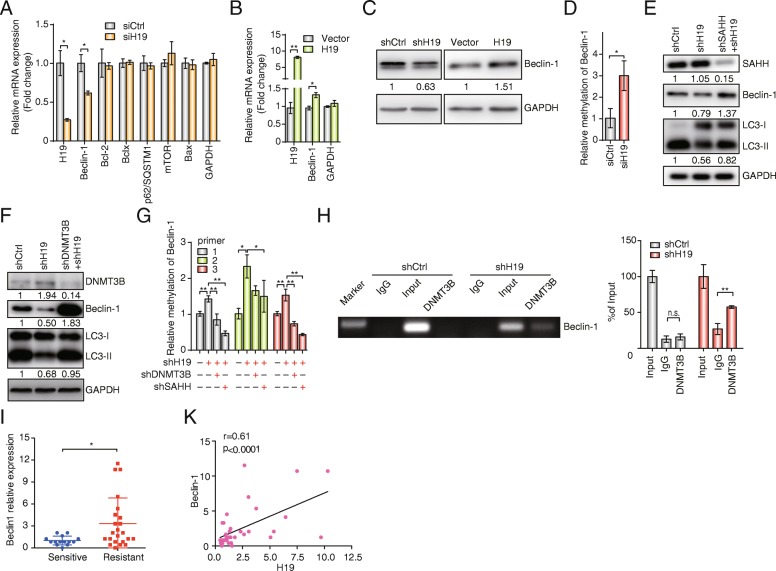
Beclin1 is regulated by H19 lncRNA *via* the H19/SAHH/DNMT3B axis. **a** The effects of H19 knockdown on the mRNA expression levels of autophagy-related genes in MCF7/TAMR cells were analyzed by qRT-PCR. The data are presented as the mean ± SD of three independent experiments. Student’s *t* test was used for statistical analysis. **b** The mRNA expression of Beclin1 in MCF7/TAMR cells overexpressing H19 or the control vector was evaluated by qRT-PCR. The data are presented as the mean ± SD of three independent experiments. Student’s *t* test was used for statistical analysis. **c** The expression of Beclin1 in MCF7/TAMR cells with knockdown or overexpression of H19 was analyzed by Western blotting. The value next to each blot is the quantification of the relative expression of the indicated band normalized to GAPDH expression. **d** DNA methylation in the promoter region of Beclin1 in MCF7/TAMR cells that stably expressed shControl (shCtrl) or shH19 was determined by real-time quantitative methylation-specific polymerase chain reaction (QMSP). The data are presented as the mean ± SD of three independent experiments. Student’s *t* test was used for statistical analysis. **e** The expression of SAHH, Beclin1, and LC3 in MCF7/TAMR cells with single H19 knockdown or double knockdown of H19/SAHH was analyzed by Western blotting. The value next to each blot is the quantification of the relative expression of the indicated band normalized to GAPDH expression. **f** The expression of DNMT3B, Beclin1, and LC3 in MCF7/TAMR cells with single H19 knockdown or double knockdown of H19/DNMT3B was analyzed by Western blotting. The value next to each blot is the quantification of the relative expression of the indicated band normalized to GAPDH expression. **g** DNA methylation in the promoter region of Beclin1 in MCF7/TAMR cells that stably expressed shCtrl, shH19, shH19+shSAHH, or shH19+shDNMT3B was determined by QMSP. The data are presented as the mean ± SD of three independent experiments. Student’s *t* test was used for statistical analysis. **h** The binding of DNMT3B to the promoter region of the Beclin1 gene was determined by a ChIP assay. The input was used as a positive control, and normal rabbit IgG was used as a negative control. The data are presented as the mean ± SD of three independent experiments. Student’s t-test was used for statistical analysis. **i** mRNA expression of Beclin1 in 37 breast cancer tissue samples. The data are presented as the mean ± SD; *n* = 37. The Wilcoxon signed-rank test was used for statistical analysis. **k** Spearman correlation analysis of H19 and Beclin1 expression in 37 breast cancer tissue samples. Spearman correlation coefficients and *P* values were calculated. *P < 0.05, **P < 0.01 compared with the control group. n.s. indicates no significant difference

To determine the mechanism by which H19 regulates autophagy, we used six autophagy-related genes as hypothetical H19 targets [37, 38]. Among these six genes, Beclin1 showed a positive correlation with H19 regulation (Fig. 4a, b). The protein expression level of Beclin1 decreased consistently with H19 knockdown and increased with H19 overexpression (Fig. 4c and Additional file 1: Figure S3a).

It has been demonstrated that H19 binds and inhibits S-adenosylhomocysteine hydrolase (SAHH), which consequently decreases DNMT3B-mediated methylation [39]. To determine the mechanism by which H19 regulates Beclin1 expression, we examined methylation in the promoter region of Beclin1. Interestingly, we found an increase in methylation in the promoter region of Beclin1 by knocking down H19 in MCF7/TAMR cells (Fig. 4d). Next, to determine whether the H19-SAHH-DNMT3B axis is responsible for this increased methylation, double knockdown groups (H19+SAHH and H19+DNMT3B) were used. At the mRNA level, the silencing efficiency of SAHH and DNMT3B were 75% and 90%, respectively (Additional file 1: Figure S3b). Western blot analysis indicated a recovery of Beclin1 expression in the H19/SAHH and H19/DNMT3B double knockdown groups compared with that in the MCF7/TAMR-shH19 group (Fig. 4e, f). In addition, the expression of LC3-II was also reversed in the H19/SAHH and H19/DNMT3B double knockdown groups (Fig. 4e, f). We observed the rescue of Beclin1 promoter methylation levels in the two double knockdown groups (Fig. 4g). To further examine the mechanism of Beclin1 methylation, we performed a chromatin immunoprecipitation (ChIP) assay and found that DNMT3B binds to the Beclin1 promoter. Upon H19 knockdown, the amount of immunoprecipitated DNA from the Beclin1 promoter increased, indicating that DNMT3B directly binds to regions of the Beclin1 promoter and that H19 knockdown promotes this interaction (Fig. 4h).

We also examined Beclin1 mRNA expression in clinical breast cancer tissue samples, which indicated that Beclin1 expression was significantly greater in the tamoxifen-resistant group than that in the tamoxifen-sensitive group (Fig. 4i). And H19 expression positively correlated with Beclin1 expression (*P* < 0.0001; *r* = 0.61; *n* = 37) (Fig. 4k). Similar positive correlation between H19 and Beclin1 expression was also found based on the GSE28645 (Additional file 1: Figure S3d). In addition, DNA methylation in the promoter region of Beclin1 in tamoxifen-resistant cancer cells was less than that in tamoxifen-sensitive cancer cells (Additional file 1: Figure S3c).

### The H19/SAHH/DNMT3B axis is involved in tamoxifen resistance *via* autophagy

**Fig. 5:**
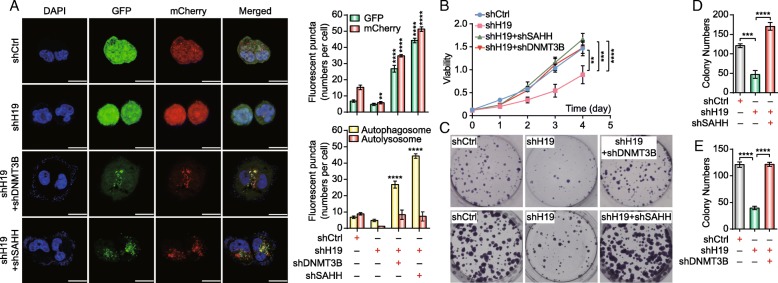
Tamoxifen resistance is regulated by the H19/SAHH/DNMT3B axis. **a** The distribution of autophagic vacuoles containing mCherry-EGFP-LC3 in MCF7/TAMR cells that stably expressed shControl (shCtrl), shH19, shH19+shSAHH, or shH19+shDNMT3B was analyzed by confocal microscopy. The bar graphs indicate the quantification of the fluorescent puncta data. The data are presented as the mean ± SD of three experiments. The scale bars indicate 20 μm. Two-way ANOVA was used for statistical analysis. **b** Under 10 μM tamoxifen treatment, the viability of MCF7/TAMR cells that stably expressed shCtrl, shH19, shH19+shSAHH, or shH19+shDNMT3B was analyzed using a CCK-8 assay. The data are presented as the mean ± SD of three independent experiments. Student’s *t* test was used for statistical analysis. **c** Representative images of the colony formation assays using MCF7/TAMR cells stably expressing shCtrl, shH19, shH19+shSAHH, or shH19+shDNMT3B under 10 μM tamoxifen treatment. **d** Bar graphs showing the quantification of the colony formation assays using MCF7/TAMR cells stably expressing shCtrl, shH19, or shH19+shSAHH. The data are presented as the mean ± SD of three independent experiments. Student’s *t* test was used for statistical analysis. **e** Bar graphs showing the quantification of the colony formation assays using MCF7/TAMR cells stably expressing shCtrl, shH19, or shH19+shDNMT3B. The data are presented as the mean ± SD of three independent experiments. Student’s *t* test was used for statistical analysis. **P < 0.01, ***P < 0.001, and ****P < 0.0001 compared with the control group

We used confocal microscopy to analyze autophagy. The two double knockdown groups (MCF7/TAMR-shH19+shSAHH and MCF7/TAMR-shH19+shDNMT3B) showed increased green and red fluorescence signals compared with the H19 knockdown group (MCF7/TAMR-shH19) (Fig. 5a). These data indicate that both SAHH and DNMT3B are involved in autophagy regulation. Moreover, to investigate whether SAHH and DNMT3B influence tamoxifen sensitivity, we performed a monolayer colony formation assay and a CCK-8 assay. Using a CCK-8 assay, we found that MCF7/TAMR-shH19+shSAHH and MCF7/TAMR-shH19+shDNMT3B cells proliferated much more rapidly than MCF7/TAMR-shH19 cells (Fig. 5b). Furthermore, compared with MCF7/TAMR-control cells, both MCF7/TAMR-shH19+shSAHH and MCF7/TAMR-shH19+shDNMT3B double knockdown groups formed more and larger colonies, while the MCF7/TAMR-shH19 cells formed fewer and smaller colonies (Fig. 5c–e). Collectively, these results indicated that H19 regulates autophagy *via* the H19-SAHH-DNMT3B axis, which further affects resistance to tamoxifen in MCF7/TAMR cells.

### Knockdown of H19 inhibits autophagy and overcomes tamoxifen resistance in vivo

**Fig. 6:**
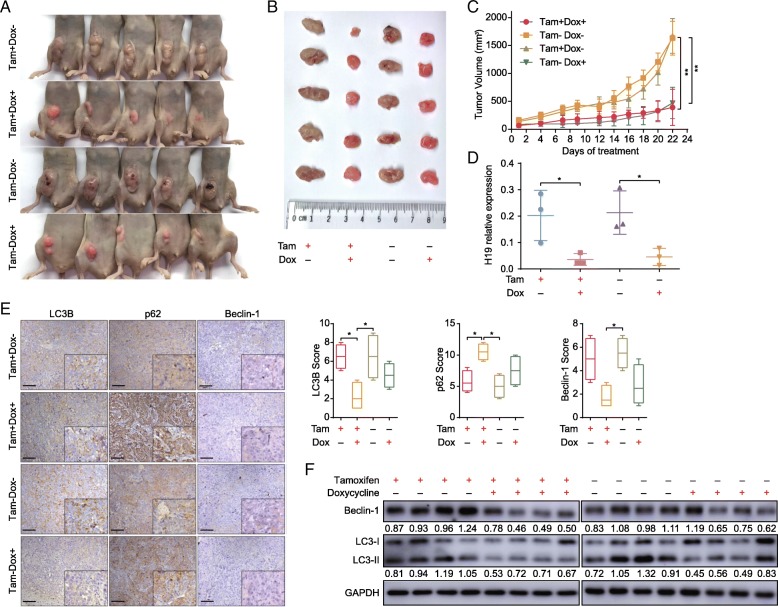
Knockdown of H19 lncRNA suppresses autophagy and restores tamoxifen sensitivity in vivo. **a**–**c** The effect of H19 expression on in vivo tumorigenicity was evaluated using a xenograft nude mouse model. Tumors formed in nude mice (**a**), isolated subcutaneous tumors (**b**), and tumor growth curves (**c**); *n* = 5 mice/group. The error bars indicate the SD. Student’s *t* test was used for statistical analysis. **d** mRNA expression of H19 in the xenografts was analyzed by qRT-PCR. The error bars indicate the SD. Student’s *t* test was used for statistical analysis. **e** Representative images of immunohistochemical (IHC) staining for Beclin1, LC3, and P62 in the xenografts. The box graphs show the quantification of IHC staining. Scale bars 50 μm. Chi-square test was used for statistical analysis. **f** Western blotting was conducted to analyze the protein expression levels of Beclin1 and LC3 in xenograft tumors. The value next to each blot is the quantification of the relative expression of the indicated band normalized to GAPDH expression. *P < 0.05, **P < 0.01 compared with the control group

To further investigate whether H19 affects the sensitivity of xenograft tumors to tamoxifen in vivo, we established xenograft tumors in nude mice by subcutaneously injecting MCF7/TAMR/Tet sh-H19 cells, in which stable H19 knockdown was induced by administering 2 mg/mouse of doxycycline (Dox) (Additional file 1: Figure S4a). The growth of tumors in groups that received Dox was significantly inhibited compared with those without Dox (Fig. 6a–c; Additional file 1: Figure S4b). The H19 expression levels of xenograft tumors in groups receiving Dox (Tam+Dox+, Tam−Dox+) were significantly less than those in the groups without Dox (Tam + Dox−, Tam−Dox−) (Fig. 6d). Immunohistochemistry (IHC) analysis indicated decreased levels of Beclin1 and LC3 but an increase in the P62 level in tumors receiving Dox compared with tumors without induction by Dox (Fig. 6e). The Western blot results also consistently showed a significant decrease in Beclin1 and LC3 protein expression levels in groups receiving Dox (Tam+Dox+, Tam−Dox+) compared with groups without Dox (Tam+Dox−, Tam−Dox−) (Fig. 6f).

## Discussion

**Fig. 7:**
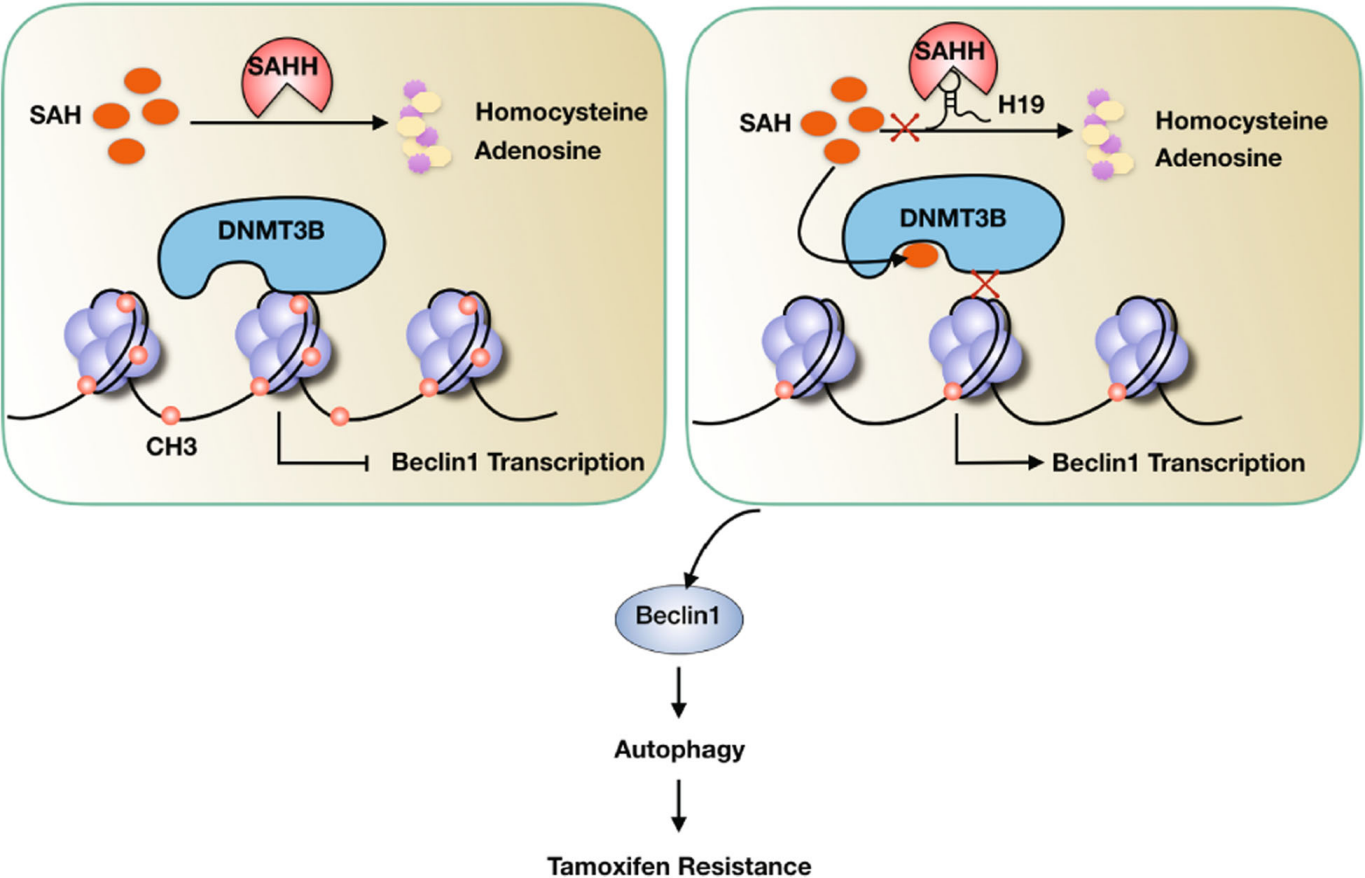
A schematic showing the involvement of H19 in tamoxifen resistance in breast cancer. When H19 is absent (left panel), SAHH hydrolyzes SAH, which causes DNMT3B-facilitated methylation in the promoter region of Beclin1; however, when H19 inhibits SAHH (right panel), it results in the accumulation of SAH, which restricts DNMT3B from methylating the promoter region of Beclin1. The increased expression level of Beclin1 subsequently initiates autophagy, conferring tamoxifen resistance in ER-positive breast cancer

Despite receiving tamoxifen treatment, one third of breast cancer patients still relapse because of developing tamoxifen resistance, which remains a major challenge in ER-positive breast cancer therapy [40]. Therefore, further investigation of tamoxifen resistance mechanisms is urgently warranted. In this study, we investigated a novel model in which upregulation of H19 expression enhances autophagy *via* downregulation of the Beclin1 methylation level, resulting in tamoxifen resistance (Fig. 7).

Recent studies have shown that lncRNAs contribute to tamoxifen resistance [20, 21], but little is known about the mechanisms by which lncRNAs regulate tamoxifen resistance in breast cancer. H19 has been identified as an imprinting lncRNA and shares the same enhancer with a neighboring gene, igf2. It involves many oncogenic processes, such as tumor cell proliferation and metastasis. During tumor cell proliferation, the transcription factor E2F induces H19 expression by binding its promoter, thereby accelerating the G1-S transition and the cell cycle [41]. Additionally, H19 also participates in tumor metastasis, which includes two converse events, EMT and MET [42, 43]. H19 also acts as a molecular sponge for microRNA let-7, regulating tumor metastasis [44]. Furthermore, significant overexpression of H19 has been observed in either ductal carcinoma in situ (DCIS) or invasive breast cancer (IBC) compared with H19 expression in normal breast tissues (*P* < 0.05) [45]. In this study, we revealed that the expression of H19 is substantially upregulated in tamoxifen-resistant breast cancer cell line and tumor tissues, and silencing H19 sensitizes MCF7/TAMR cells to tamoxifen treatment both in vitro and in vivo. Recent studies have reported that ER promotes H19 expression, and H19 is upregulated in ER^+^ breast cancer [28, 46]. Because tamoxifen-resistant breast cancers rarely harbor ESR1 mutations, which confer only partial resistance to tamoxifen [47–50], the upstream molecular regulator of H19 in developing tamoxifen resistance remains to be investigated.

Although in some settings autophagy inhibits tumorigenesis, in most conditions, autophagy promotes cancer initiation and progression [51]. Tumors enhance autophagy activity to survive microenvironmental stress and to facilitate proliferation and aggressiveness by suppressing stress responses and promoting metabolism and survival [51–53]. Recently, dysregulations in autophagy function have been proved to be a potential mechanism of developing tamoxifen-resistant breast cancer [54]. H19 has also been found to regulate autophagy, but the results have not been consistent thus far. For instance, in a rat brain with cerebral ischemia and reperfusion injury, H19 has been shown to activate the autophagy process through the DUSP5-ERK1/2 axis [55]. However, Zhuo et al. found that H19 inhibits autophagy progression in cardiomyocytes by silencing DIRAS3 epigenetically [56]. In this study, we elucidate how H19 regulates autophagy in the setting of tamoxifen-resistant breast cancer.

Recent studies have indicated that epigenetic modifications, such as DNA methylation, play important roles in regulating autophagy [57–59]. S-adenosylhomocysteine hydrolase (SAHH) has been reported as the only mammalian enzyme to hydrolyze S-adenosylhomocysteine (SAH). In our previous work, we demonstrated the H19/SAHH/DNMT3B axis, in which the direct binding of H19 to SAHH in mouse myoblasts inhibits the downstream hydrolysis of SAH, leading to the genome-wide inhibition of DNA methyltransferase 3B (DNMT3B)-mediated methylation [39]. In this study, we demonstrated that the expression level of Beclin1 significantly decreases by silencing H19 and increases by overexpressing H19. Then, in both double knockdown groups (H19+SAHH or H19+DNMT3B), a recovery of Beclin1 expression was observed compared with Beclin1 expression in the H19 knockdown group. The ChIP assay validated that silencing H19 enhances the interaction of DNMT3B with the Beclin1 promoter region, thus decreasing the expression level of Beclin1 and attenuating the activity of autophagy. Therefore, we propose a novel mechanism by which H19 regulates autophagy *via* changes in DNA methylation.

In the clinical setting, lncRNAs have great potential as prognostic or diagnostic indicators [19, 60] because of their cancer-restricted expression characteristics and excellent stability in biological fluids. The lncRNA HOTAIR is upregulated in primary breast cancers and metastases, and the HOTAIR expression level in primary cancers also serves as a promising predictor of eventual metastasis and death [19]. Antisense oligonucleotides (ASOs), which direct RNase H to cleave complementary target lncRNA, potently decrease lncRNA function [61]. ASO has been used to downregulate MALAT1 lncRNA expression in mouse mammary tumor virus-PyMT mice and organoids, resulting in decreases in tumor cell proliferation and metastasis [62]. In this study, it was shown that the overexpression of H19 in tamoxifen-resistant breast cancer cells serves as a promising predictive biomarker for clinical patients. The preclinical xenograft tumor assays in nude mice indicated that H19 could be a potential target to reverse tamoxifen resistance. Further research and technical innovation regarding the inhibition of H19 could provide opportunities for H19-targeted therapies in tamoxifen-resistant breast cancer.

## Conclusions

In summary, we demonstrated that upregulation of H19 expression enhances autophagy and ultimately leads to tamoxifen resistance in ER-positive breast cancer cells by decreasing methylation in the promoter region of Beclin1 *via* the H19/SAHH/DNMT3B axis. We propose that H19 serves as a potential therapeutic target for the treatment of patients with ER-positive breast cancer.

## Additional files

Additional file 1: **Figure S1.** Inhibition of autophagy affects tamoxifen resistance. **Figure S2.** Inhibition of H19 suppresses resistance to tamoxifen. **Figure S3.** H19 positively regulates Beclin1. **Figure S4.** Silencing H19 lncRNA inhibits resistance to tamoxifen in vivo. (DOCX 2570 kb)

Additional file 2: Supplementary tables. This file contains supplementary Tables S1 and S2. (DOCX 517 kb)

## Data Availability

The datasets used and/or analyzed during the current study are available from the corresponding author on reasonable request.

## Abbreviations

3-MA: 3-methyladenine
ASOs: Antisense oligonucleotides
ChIP: Chromatin immunoprecipitation
CQ: Chloroquine
DCIS: Ductal carcinoma in situ
DNMT3B: DNA methyltransferase 3B
Dox: Doxycycline
ER: Estrogen receptor
FBS: Fetal bovine serum
H19: lncRNA H19
IBC: Invasive breast cancer
IHC: Immunohistochemistry
lncRNAs: Long noncoding RNAs
MCF7/TAMR: Tamoxifen-resistant MCF7 cell line
MRI: Magnetic resonance imaging
QMSP: Quantitative methylation-specific polymerase chain reaction
SAH: S-Adenosylhomocysteine
SAHH: S-Adenosylhomocysteine hydrolase
shCtrl: shControl
shRNAs: Short hairpin RNAs
Stv: Starvation
TAM: Tamoxifen

## References

[1] Siegel RL, Miller KD, Jemal A (2018). Cancer statistics, 2018. CA Cancer J Clin.

[2] Clarke R, Liu MC, Bouker KB, Gu Z, Lee RY, Zhu Y (2003). Antiestrogen resistance in breast cancer and the role of estrogen receptor signaling. Oncogene..

[3] Davies C, Godwin J, Gray R, Clarke M, Cutter D, Early Breast Cancer Trialists’ Collaborative G (2011). Relevance of breast cancer hormone receptors and other factors to the efficacy of adjuvant tamoxifen: patient-level meta-analysis of randomised trials. Lancet..

[4] Burstein HJ, Temin S, Anderson H, Buchholz TA, Davidson NE, Gelmon KE (2014). Adjuvant endocrine therapy for women with hormone receptor-positive breast cancer: american society of clinical oncology clinical practice guideline focused update. J Clin Oncol..

[5] Dees EC, Carey LA (2013). Improving endocrine therapy for breast cancer: it’s not that simple. J Clin Oncol..

[6] Arpino G, Wiechmann L, Osborne CK, Schiff R (2008). Crosstalk between the estrogen receptor and the HER tyrosine kinase receptor family: molecular mechanism and clinical implications for endocrine therapy resistance. Endocrine reviews..

[7] Musgrove EA, Sutherland RL (2009). Biological determinants of endocrine resistance in breast cancer. Nat Rev Cancer..

[8] Klionsky DJ, Abdelmohsen K, Abe A, Abedin MJ, Abeliovich H, Acevedo Arozena A (2016). Guidelines for the use and interpretation of assays for monitoring autophagy (3rd). Autophagy..

[9] Klionsky DJ (2008). Autophagy revisited: a conversation with Christian de Duve. Autophagy..

[10] John S, Nayvelt I, Hsu HC, Yang P, Liu W, Das GM (2008). Regulation of estrogenic effects by beclin 1 in breast cancer cells. Cancer Res..

[11] Cook KL, Shajahan AN, Clarke R (2011). Autophagy and endocrine resistance in breast cancer. Expert Rev Anticancer Ther.

[12] Qadir MA, Kwok B, Dragowska WH, Le D, Bally MB, To KH (2008). Macroautophagy inhibition sensitizes tamoxifen-resistant breast cancer cells and enhances mitochondrial depolarization. Breast Cancer Res Treat..

[13] Samaddar JS, Gaddy VT, Duplantier J, Thandavan SP, Shah M, Smith MJ (2008). A role for macroautophagy in protection against 4-hydroxytamoxifen-induced cell death and the development of antiestrogen resistance. Mol Cancer Ther..

[14] Cook KL, Warri A, Soto-Pantoja DR, Clarke PA, Cruz MI, Zwart A (2014). Hydroxychloroquine inhibits autophagy to potentiate antiestrogen responsiveness in ER+ breast cancer. Clin Cancer Res.

[15] Prensner JR, Chinnaiyan AM (2011). The emergence of lncRNAs in cancer biology. Cancer Discov..

[16] Ulitsky I, Bartel DP (2013). lincRNAs: genomics, evolution, and mechanisms. Cell..

[17] Prensner JR, Iyer MK, Sahu A, Asangani IA, Cao Q, Patel L (2013). The long noncoding RNA SChLAP1 promotes aggressive prostate cancer and antagonizes the SWI/SNF complex. Nat Genet..

[18] Huarte M, Guttman M, Feldser D, Garber M, Koziol MJ, Kenzelmann-Broz D (2010). A large intergenic noncoding RNA induced by p53 mediates global gene repression in the p53 response. Cell..

[19] Gupta RA, Shah N, Wang KC, Kim J, Horlings HM, Wong DJ (2010). Long non-coding RNA HOTAIR reprograms chromatin state to promote cancer metastasis. Nature..

[20] Xue X, Yang YA, Zhang A, Fong KW, Kim J, Song B (2016). LncRNA HOTAIR enhances ER signaling and confers tamoxifen resistance in breast cancer. Oncogene..

[21] Niknafs YS, Han S, Ma T, Speers C, Zhang C, Wilder-Romans K (2016). The lncRNA landscape of breast cancer reveals a role for DSCAM-AS1 in breast cancer progression. Nat Commun..

[22] Pachnis V, Belayew A, Tilghman SM (1984). Locus unlinked to alpha-fetoprotein under the control of the murine raf and Rif genes. Proc Natl Acad Sci U S A..

[23] Zemel S, Bartolomei MS, Tilghman SM (1992). Physical linkage of two mammalian imprinted genes, H19 and insulin-like growth factor 2. Nat Genet..

[24] Raveh E, Matouk IJ, Gilon M, Hochberg A (2015). The H19 Long non-coding RNA in cancer initiation, progression and metastasis - a proposed unifying theory. Mol Cancer..

[25] Peng F, Li TT, Wang KL, Xiao GQ, Wang JH, Zhao HD (2017). H19/let-7/LIN28 reciprocal negative regulatory circuit promotes breast cancer stem cell maintenance. Cell Death Dis..

[26] Zhu QN, Wang G, Guo Y, Peng Y, Zhang R, Deng JL (2017). LncRNA H19 is a major mediator of doxorubicin chemoresistance in breast cancer cells through a cullin4A-MDR1 pathway. Oncotarget..

[27] Zhou Wu, Ye Xiao-lei, Xu Jun, Cao Ming-Guo, Fang Zheng-Yu, Li Ling-Yun, Guan Guang-Hui, Liu Qiong, Qian Yue-Hui, Xie Dong (2017). The lncRNA H19 mediates breast cancer cell plasticity during EMT and MET plasticity by differentially sponging miR-200b/c and let-7b. Science Signaling.

[28] Sun H, Wang G, Peng Y, Zeng Y, Zhu QN, Li TL (2015). H19 lncRNA mediates 17beta-estradiol-induced cell proliferation in MCF-7 breast cancer cells. Oncol Rep..

[29] Basak P, Chatterjee S, Weger S, Bruce MC, Murphy LC, Raouf A (2015). Estrogen regulates luminal progenitor cell differentiation through H19 gene expression. Endocr Relat Cancer.

[30] Li G, Zhang J, Jin K, He K, Zheng Y, Xu X (2013). Estrogen receptor-alpha36 is involved in development of acquired tamoxifen resistance via regulating the growth status switch in breast cancer cells. Mol Oncol.

[31] Jia Y, Zhou J, Luo X, Chen M, Chen Y, Wang J (2018). KLF4 overcomes tamoxifen resistance by suppressing MAPK signaling pathway and predicts good prognosis in breast cancer. Cell Signal..

[32] Tan D, Wu Y, Hu L, He P, Xiong G, Bai Y (2017). Long noncoding RNA H19 is up-regulated in esophageal squamous cell carcinoma and promotes cell proliferation and metastasis. Dis Esophagus.

[33] Sowinska A, Jagodzinski PP (2007). RNA interference-mediated knockdown of DNMT1 and DNMT3B induces CXCL12 expression in MCF-7 breast cancer and AsPC1 pancreatic carcinoma cell lines. Cancer Lett.

[34] Gao H, Hao G, Sun Y, Li L, Wang Y (2018). Long noncoding RNA H19 mediated the chemosensitivity of breast cancer cells via Wnt pathway and EMT process. OncoTargets Ther.

[35] Gonzalez-Malerva L, Park J, Zou L, Hu Y, Moradpour Z, Pearlberg J (2011). High-throughput ectopic expression screen for tamoxifen resistance identifies an atypical kinase that blocks autophagy. Proc Natl Acad Sci U S A..

[36] Walsh CA, Bolger JC, Byrne C, Cocchiglia S, Hao Y, Fagan A (2014). Global gene repression by the steroid receptor coactivator SRC-1 promotes oncogenesis. Cancer Res..

[37] Levy JMM, Towers CG, Thorburn A (2017). Targeting autophagy in cancer. Nat Rev Cancer..

[38] Doherty J, Baehrecke EH (2018). Life, death and autophagy. Nat Cell Biol.

[39] Zhou J, Yang L, Zhong T, Mueller M, Men Y, Zhang N (2015). H19 lncRNA alters DNA methylation genome wide by regulating S-adenosylhomocysteine hydrolase. Nat Commun..

[40] Early Breast Cancer Trialists' Collaborative G (2005). Effects of chemotherapy and hormonal therapy for early breast cancer on recurrence and 15-year survival: an overview of the randomised trials. Lancet..

[41] Berteaux N, Lottin S, Monte D, Pinte S, Quatannens B, Coll J (2005). H19 mRNA-like noncoding RNA promotes breast cancer cell proliferation through positive control by E2F1. J Biol Chem.

[42] Tsang WP, Ng EK, Ng SS, Jin H, Yu J, Sung JJ (2010). Oncofetal H19-derived miR-675 regulates tumor suppressor RB in human colorectal cancer. Carcinogenesis..

[43] Keniry A, Oxley D, Monnier P, Kyba M, Dandolo L, Smits G (2012). The H19 lincRNA is a developmental reservoir of miR-675 that suppresses growth and Igf1r. Nat Cell Biol..

[44] Yan L, Zhou J, Gao Y, Ghazal S, Lu L, Bellone S (2015). Regulation of tumor cell migration and invasion by the H19/let-7 axis is antagonized by metformin-induced DNA methylation. Oncogene..

[45] Zhang Z, Weaver DL, Olsen D, deKay J, Peng Z, Ashikaga T (2016). Long non-coding RNA chromogenic in situ hybridisation signal pattern correlation with breast tumour pathology. J Clin Pathol..

[46] Basak P, Chatterjee S, Bhat V, Su A, Jin H, Lee-Wing V (2018). Long non-coding RNA H19 acts as an estrogen receptor modulator that is required for endocrine therapy resistance in ER+ breast cancer cells. Cell Physiol Biochem.

[47] Jeselsohn R, Yelensky R, Buchwalter G, Frampton G, Meric-Bernstam F, Gonzalez-Angulo AM (2014). Emergence of constitutively active estrogen receptor-alpha mutations in pretreated advanced estrogen receptor-positive breast cancer. Clin Cancer Res.

[48] Fribbens C, O'Leary B, Kilburn L, Hrebien S, Garcia-Murillas I, Beaney M (2016). Plasma ESR1 mutations and the treatment of estrogen receptor-positive advanced breast cancer. J Clin Oncol..

[49] Schiavon G, Hrebien S, Garcia-Murillas I, Cutts RJ, Pearson A, Tarazona N (2015). Analysis of ESR1 mutation in circulating tumor DNA demonstrates evolution during therapy for metastatic breast cancer. Sci Transl Med.

[50] Nayar U, Cohen O, Kapstad C, Cuoco MS, Waks AG, Wander SA (2019). Acquired HER2 mutations in ER(+) metastatic breast cancer confer resistance to estrogen receptor-directed therapies. Nat Genet..

[51] White E (2015). The role for autophagy in cancer. J Clin Invest.

[52] Strohecker AM, Guo JY, Karsli-Uzunbas G, Price SM, Chen GJ, Mathew R (2013). Autophagy sustains mitochondrial glutamine metabolism and growth of BrafV600E-driven lung tumors. Cancer Discov..

[53] Guo JY, Chen HY, Mathew R, Fan J, Strohecker AM, Karsli-Uzunbas G (2011). Activated Ras requires autophagy to maintain oxidative metabolism and tumorigenesis. Genes Dev.

[54] Lee MH, Koh D, Na H, Ka NL, Kim S, Kim HJ (2018). MTA1 is a novel regulator of autophagy that induces tamoxifen resistance in breast cancer cells. Autophagy..

[55] Wang J, Cao B, Han D, Sun M, Feng J (2017). Long non-coding RNA H19 induces cerebral ischemia reperfusion injury via activation of autophagy. Aging Dis.

[56] Zhuo C, Jiang R, Lin X, Shao M (2017). LncRNA H19 inhibits autophagy by epigenetically silencing of DIRAS3 in diabetic cardiomyopathy. Oncotarget..

[57] Sutter BM, Wu X, Laxman S, Tu BP (2013). Methionine inhibits autophagy and promotes growth by inducing the SAM-responsive methylation of PP2A. Cell..

[58] Bai H, Inoue J, Kawano T, Inazawa J (2012). A transcriptional variant of the LC3A gene is involved in autophagy and frequently inactivated in human cancers. Oncogene..

[59] Hu X, Sui X, Li L, Huang X, Rong R, Su X (2013). Protocadherin 17 acts as a tumour suppressor inducing tumour cell apoptosis and autophagy, and is frequently methylated in gastric and colorectal cancers. J Pathol.

[60] Tan DSW, Chong FT, Leong HS, Toh SY, Lau DP, Kwang XL (2017). Long noncoding RNA EGFR-AS1 mediates epidermal growth factor receptor addiction and modulates treatment response in squamous cell carcinoma. Nat Med..

[61] Ideue T, Hino K, Kitao S, Yokoi T, Hirose T (2009). Efficient oligonucleotide-mediated degradation of nuclear noncoding RNAs in mammalian cultured cells. RNA (New York, NY)..

[62] Arun G, Diermeier S, Akerman M, Chang KC, Wilkinson JE, Hearn S (2016). Differentiation of mammary tumors and reduction in metastasis upon Malat1 lncRNA loss. Genes Dev.

