# Supplementary material for: The long noncoding RNA H19 promotes tamoxifen resistance in breast cancer via autophagy
Source: J Hematol Oncol. 2019 Jul 24;12:81. doi: 10.1186/s13045-019-0747-0 (PMC6657081; doi:10.1186/s13045-019-0747-0)
Supplement: Supplementary file 1 — Figure S1. Inhibition of autophagy affects tamoxifen resistance. Figure S2. Inhibition of H19 suppresses resistance to tamoxifen. Figure S3. H19 positively regulates Beclin1. Figure S4. Silencing H19 lncRNA inhibits resistance to tamoxifen in vivo. (DOCX 2570 kb) [file 13045_2019_747_MOESM1_ESM.docx]

**Supplementary Materials for**

**The long noncoding RNA H19 promotes tamoxifen resistance in breast cancer *via* autophagy**

**Additional file 1
Supplementary Figure legends**

**Figure S1. Inhibition of autophagy affects tamoxifen resistance.**

a. MCF7 or MCF7/TAMR cells cultured in different concentrations of tamoxifen were analyzed using a CCK-8 assay. The data were then used to calculate the IC50 of each cell line. The data are presented as the mean ± SD of three independent experiments.

**Figure S2. Inhibition of H19 suppresses resistance to tamoxifen.**

a. Under 10 μM tamoxifen treatment, the viability of MCF-7/TAMR cells that stably expressed shH19 or shCtrl was analyzed using a CCK-8 assay. The data are presented as the mean ± SD of three independent experiments.

b. MCF7 tamoxifen-sensitive cells transfected with siCtrl or siH19 were cultured in different concentrations of tamoxifen, and were analyzed using a CCK-8 assay. The data were then used to calculate the IC50 of each cell line. The data are presented as the mean ± SD of three independent experiments.

**Figure S3. H19 positively regulates Beclin1.**

a. The expression of Beclin1 in MCF7/TAMR cells with siRNA knockdown of H19 was analyzed by western blotting. The value next to each blot is the quantification of the relative expression of the indicated band normalized to GAPDH expression.

b. MCF7/TAMR cells were infected with shCtrl, shH19 and/or shSAHH or shDNMT3B lentiviral vectors. The efficiency of RNA interference-mediated knockdown of target gene expression was determined by RT-PCR analysis. The data are presented as the mean ± SD of three independent experiments.

c. DNA methylation in the promoter region of Beclin1 in cells from clinical tissue samples was evaluated by real-time quantitative methylation-specific polymerase chain reaction (QMSP). The data are presented as the mean ± SD of three independent experiments.

d. Spearman correlation analysis of H19 and Beclin1 expression in breast cancer cell line based on GSE28645. Spearman correlation coefficients and *P* values were calculated.

**Figure S4. Silencing H19 lncRNA** **inhibits resistance to tamoxifen *in vivo*.**

a. To demonstrate the effectiveness of Dox-induced H19 knockdown, we cultured cells in medium with different concentrations of Dox for 48 hours. qRT-PCR was used to evaluate the expression of H19. The data are presented as the mean ± SD of three independent experiments. b. Representative images of magnetic resonance imaging (MRI) showing the size of the xenografts. The images are shown in transverse and coronal views. n=5 mice/group.

* P < 0.05, ** P < 0.01, *** P < 0.001, **** P < 0.0001 compared with the control group.
