# Supplementary material for: The long noncoding RNA H19 promotes tamoxifen resistance in breast cancer via autophagy
Source: J Hematol Oncol. 2019 Jul 24;12:81. doi: 10.1186/s13045-019-0747-0 (PMC6657081; doi:10.1186/s13045-019-0747-0)
Supplement: Supplementary file 2 — Supplementary tables. This file contains supplementary Tables S1 and S2. (DOCX 517 kb) [file 13045_2019_747_MOESM2_ESM.docx]

| Supplementary Table 1. QMSP primer sequences | | |
| --- | --- | --- |
| Gene | Forward primer | Reverse primer |
| Beclin1-primer1 | 5'-TTTAAAGTGTTGGAATTATAAGCGT-3' | 5'-TCGAAAAACTAAAACAAAAAAATCG-3' |
| Beclin1-primer2 | 5'-TTCGAATTTTTGATTTTGTGATTC-3' | 5'-ACCACTACACTCCAACCTAAACG-3' |
| Beclin1-primer3 | 5'-AGTTCGGAGTTCGTTATTTAAGTTC-3' | 5'-GAAAAATCGCTAAAAACAAAACG-3' |
| Albumin | 5'-GTGAGAATTGTAGAGCAGTGCTGTC-3' | 5'-ACATTGCTCAGCACAGATCCAC-3' |

| Supplementary Table 2. Real-time PCR primer sequences | | |
| --- | --- | --- |
| Gene | Forward primer | Reverse primer |
| H19 | 5'-ACTCAGGAATCGGCTCTGGAA-3' | 5'-CTGCTGTTCCGATGGTGTCTT-3' |
| Beclin1 | 5'-GGCTGAGAGACTGGATCAGG-3' | 5'-CTGCGTCTGGGCATAACG-3' |
| SAHH | 5'-CAACATGATTCTGGACGACG-3' | 5'-GACATTGATGGCAGGCAC-3' |
| DNMT3B | 5'-GGCAAGTTCTCCGAGGTCTCTG-3' | 5'TGGTACATGGCTTTTCGATAGGA-3' |
| β-Tubulin | 5'-CGTGTTCGGCCAGAGTGGTGC-3' | 5'-GGGTGAGGGCATGACGCTGAA-3' |
| β-Actin | 5’-CTGGAACGGTGAAGGTGACA-3’ | 5’-AAGGGACTTCCTGTAACAATGCA-3' |
